# Effects of starch concentration on calcium‐enhanced black bullhead catfish protein gels

**DOI:** 10.1002/fsn3.456

**Published:** 2017-01-22

**Authors:** Ilgin Paker, Kristen E. Matak

**Affiliations:** ^1^ Food Engineering DepartmentYeditepe UniversityKayisdagiIstanbulTurkey; ^2^ West Virginia UniversityAnimal and Nutritional SciencesPO Box 6108MorgantownWest VirginiaUSA

**Keywords:** Differential scanning calorimetry, protein gel, starch, texture profile analysis

## Abstract

Calcium‐enhanced protein recovered from black bullhead catfish was used to develop gels containing increasing amounts of potato starch (0–20 g/kg protein paste) and the effects of starch on functional, textural, and color properties were tested. Energy required to unfold protein groups was greater with the addition of 5 g starch/kg protein paste. Gels containing starch were harder, chewier, and less springy (*p* < .05) than gels without starch. For most measurements, regression analysis showed that increasing the starch concentration beyond 5 g/kg did not contribute to further significant textural changes. Torsional shear stress and strain along with Kramer shear force increased as the concentration of starch increased (*R* ^*2*^ = .79, .79, and .53, respectively). The addition of ≥10 g starch/kg protein paste resulted in darker gels and gels got darker as more starch was added (*R* ^*2*^ = .71). Results showed no benefit to increasing starch concentration in gels beyond 5 g starch/kg protein paste.

## 1 Introduction

Myofibrillar protein consists of myosin and actin and form thermally induced protein gels by establishing a three‐dimensional network between protein paste constituents such as fat, protein, and water (Brewer, Peterson, Carr, Mccusker, & Novakofski, 2005; Sun & Holley, 2011). The structural and textural properties of the final gel depend on the functionality of the protein groups as well as the composition of the gel formula such as the salt amount, myosin to actin ratio, and other ingredients that might interfere with protein unfolding and aggregation. Functional additives such as starch, salt, polyphosphates, and transglutaminase (an exogenous enzyme) are often used when making protein gels to enhance gelation by increasing the intermolecular interactions, water‐holding capacity, and rate of cross‐linking (Brewer et al., 2005; Sun & Holley, 2011).

Starch granules absorb water and swell during gel formation (Lee, Wu, & Okada, 1992). Upon heating, they interact with protein and gelatinize, thus increasing gel strength (Hunt, Getty, & Park, 2010). Amylose to amylopectin ratio influences the gelation properties of different starches, with amylopectin the main contributor to gel network formation. For example, potato starch has a greater concentration of amylopectin than wheat starch and therefore produces firmer and more cohesive gels (Kim & Lee, 1987). Gel‐strengthening properties of starch would be especially beneficial for protein gels made from chemically processed protein, such as proteins recovered using pH shift processes, or proteins containing nutritive additives, such as sarcoplasmic proteins, that may affect gel formation (Paker & Matak, 2015). Therefore, the aim of this study was to assess the impact of increasing amounts of potato starch (0, 5, 10, 15, 20 g/kg protein paste) on the functional, textural, and color properties of calcium‐enhanced myofibrillar protein gels.

## 2 Materials and Methods

### 2.1 Ground catfish preparation

All equipment used to handle fish were sanitized prior to fish arrival. Freshly caught (Dog Wood Lake, Morgantown WV) black bullhead catfish (*Ameiurus melas*) were transported to the meat‐processing laboratory at West Virginia University in containers with ice and carbon monoxide within 1 h of being caught. Fish were rinsed under running tap water, gutted, and coarsely ground twice using a meat processor (Hobart Model 4146, Troy, OH) with a coarse grinding plate attachment. The coarse fish paste was transferred to lidded stainless steel trays for storage at −20°C for 12 h to prevent thermal denaturation. Chilled coarse fish paste was twice processed through the meat grinder using a fine grinder plate. The finely ground fish were individually packaged (500 g) into freezer bags (Ziplock Freezer Bags, S.C. Johnson & Son, Inc., Racine, WI), vacuum packaged (Ultravac KOCH Packaging, KOCH Supplies Inc., Kansas City, MO), and stored at −80°C until analyses were conducted.

### 2.2 Myofibrillar protein separation using ISP

Myofibrillar fish protein was separated from the other components of the fish paste using a method called isoelectric solubilization and precipitation (ISP) that relies on pH shifts to solubilize and then precipitate the protein (Gehring, Gigliotti, Moritz, Tou, & Jaczynski, 2011). Thawed (4°C for 24–48 h) ground catfish (500 g) was mixed with distilled deionized water at a 1:6 (fish:water) ratio in a glass beaker. Protein extraction was carried out as described elsewhere (Paker, Beamer, Jaczynski, & Matak, 2013b). The initial pH of the mixture was pH 6.6 ± 0.2, confirmed using a calibrated pH/ion analyzer (Oakton, Eutech Instruments; Singapore). Protein solubilization was facilitated with the addition of 1 mol L^−1^ calcium hydroxide (Ca(OH)_2_) to the mixture to raise to the pH to 11.0 while homogenizing using a laboratory grade, sanitized homogenizer (PowerGen 700, Fisher Scientific, Pittsburgh, PA) (Paker, Beamer, Jaczynski, & Matak, 2013a). After reaching the targeted pH value, homogenization continued for an additional 10 min and afterward the mixture was centrifuged at 10,000*g* for 15 min at 4°C (Sorvall RC‐SB Refrigerated Superspeed Centrifuge, Du Pont, Wilmington, DE). Distinct layers formed with centrifugation, with lipids on the top layer and insoluble components (such as skin, bones, and scales) on the bottom. The solubilized protein in the aqueous middle layer was filtered through a cheesecloth into a glass beaker. During homogenization, pH was reduced to the protein isoelectric point of pH 5.5 with the addition of glacial l‐lactic acid (85%, EMD Chemicals, Netherlands). The mixture was homogenized for an additional 10 min after reaching the target pH to allow ample time for precipitation. The solution was then centrifuged at 10,000*g* for 15 min at 4°C and the protein fraction that formed a pellet at the bottom of the centrifuge tubes was collected and the supernatant discarded. The recovered protein was used for paste development immediately after collection.

### 2.3 Protein paste development

Immediately following protein recovery, a protein paste was developed using the collected protein. A universal food processor (model UMC5, Stephan Machinery Corp., Columbus, OH) chopped protein at low speed for 1 min, 20 g salt/kg protein paste was added, and the mixture was chopped for 1 min. The initial moisture of the recovered protein was confirmed by a moisture analyzer (Ohaus, Model MB45, Switzerland) and adjusted to 80 g/100 g by adding chilled water (4°C). Transglutaminase (5 g/kg protein paste), 3 g/kg protein paste polyphosphates (Kena FP‐28, Innophos, Cranbury, NJ) and 0, 5, 10, 15, or 20 g/kg protein paste potato starch (Penbind 1000 modified potato starch, Penford Food Ingredients Corp., Centennial, CO) was added depending on the tested formula. After the final pH of the mixture was adjusted to pH 7.0–7.2 (Oakton, Eutech Instruments; Singapore) by adding lactic acid, the mixture was chopped for 3 min at high speed under vacuum (50 kPa). Thermal denaturation was prevented by maintaining the temperature of the universal food processor at 1–4°C throughout the process. The protein paste was transferred to vacuum bags and vacuum‐packed to remove air that might interfere with the texture and color analyses (Ultravac KOCH Packaging, KOCH Supplies Inc.). A sample of protein paste was retained for differential scanning calorimetry (DSC) analysis immediately after paste development. Using a gel presser, the remaining protein paste was pressed into stainless steel tubes (length = 17.5 cm, inner diameter = 1.9 cm) and dumbbell‐shaped stainless steel torsion tubes (length = 17.5 cm, end diameter = 1.9 cm, midsection diameter = 1.0 cm) that were lightly sprayed with canola oil to avoid sticking of gels upon cooking. The tubes containing the pastes were stored in a refrigerator (4°C) for 24 h to allow for gel formation.

### 2.4 Differential scanning calorimetry analysis

Thermal changes in protein pastes (10–15 μg) were assessed using DSC (DSC Infinity Series F5010, Instrument Specialists, Inc., Spring Grove, IL) during which temperature was increased from 5°C to 90°C at a rate of 10°C min^−1^. The analysis was replicated four times per each gel sample containing different amounts of starch and the results were computed using Infinite Software (Instrument Specialists, Inc.). Thermograms were drawn for each sample using the mean value of four replications. Temperature onset, temperature maximum, and enthalpy required for thermal transactions were presented as mean ± standard deviation.

### 2.5 Protein gel development

The steel tubes containing protein pastes were removed from refrigerated storage and cooked at 90°C for 20 min in a water bath (Precision, Jouan Inc, Wincester, Virginia). The cooked gels were chilled in an ice bath for 15 min and then acclimated at room temperature (20–22°C for 1 h). The gels were removed from the steel tubes, cut and analyzed for expressible water content, texture, and color properties.

### 2.6 Expressible water content

Expressible water content was measured for each formula by folding 5 g of sample in two layers of Whatman No. 1 filter paper and centrifuging at 7500*g* for 15 min (Eppendorf, Micro‐centrifuge 5430 R with F‐35‐6‐30 rotor, Hamburg, Germany). The final weight was recorded. Expressible water content was calculated using the formula:

$$\mathrm{Expressible}\;\;\mathrm{water}\;\;(\%)=\frac{\text{Initial weight ‐ Final weight}}{\mathrm{Initial}\;\;\mathrm{weight}}\times 100$$

The test was performed in triplicates for each formula tested and the results were given as mean ± standard deviation.

### 2.7 Texture analyses

Texture profile analysis (TPA), Kramer shear cell test, and torsional analysis were used to determine differences in texture between gel formulations. TPA describes the hardness, springiness, cohesiveness, chewiness, and resilience of gels. TPA was measured using a texture analyzer (Model TA‐HDi; Texture Technologies Corp., Scarsdale, NY) with a compression plate attachment (70 mm). Gels were cut to 2.54 cm long with a diameter of 1.9 cm.

Kramer shear cell test mimics the process of cutting a food sample with a knife and is another method used to measure firmness and cohesiveness of a gel sample. A texture analyzer (Model TA‐HDi, Texture Technologies Corp) with a Kramer cell attachment cut through the samples with a five‐blade (3 mm thick and 70 mm wide) attachment. Peak force (g peak force g^−1^ gel sample) was measured at 127 mm min^−1^ crosshead speed. Shear force and shear stress were calculated by dividing the peak force by the weight of each sample, and dividing the peak force by the area of the sample (length 8 cm, diameter 1.9 cm), respectively.

Torsional shear stress and strain measure the gel strength and uniformity by twisting the sample until breakage at the mid‐section of the dumbbell‐shaped gels. Samples were glued on the top and bottom to plastic discs and a Hamman Gelometer (Gel Consultants, Raleigh, NC) twisted the samples.

Texture profile analysis, Kramer shear, and torsion analyses were conducted on each gel sample formulation at least six times. Data from TPA and Kramer shear were calculated using Texture Expert software (Texture Expert Exceed version 2.64; Stable Micro Systems, 2003, Hamilton, MA). Data from torsional analysis were calculated using Torsion Vane software (Gel Consultants).

### 2.8 Color measurement

Color was measured using a colorimeter (Minolta Camera Co. Ltd, Osaka, Japan) calibrated with a standard white plate No.21333180 (CIE L* 93.1; a* 0.3135; b* 0.3198). After collecting the data for L* (lightness; scale: 0–100), a* (intensity in red color; scale: −60 to +60), and b* (intensity in yellow color; scale: −60 to +60), whiteness was calculated using the equation:

$$\mathrm{Whiteness}=100-{\left[{\left(100-L\right)}^{2}+a^{2}+b^{2}\right]}^{1/2}$$

At least 12 samples were tested for each formula and the results were given as mean ± standard deviation.

### 2.9 Statistical analysis

ISP and formulation trials were randomized prior to beginning of the study using JMP software (JMP 10.2, SAS Inst., Cary, NC). One‐way analysis of variance (ANOVA) was used at a significance level of 0.05 and the mean values for each formula tested were statistically separated using Tukey's Honestly Significant Difference test. Data were presented as mean ± standard deviation. Different letters (^a,b,c^) were assigned to significantly different values of tested gels containing different amounts of starch. Regression analysis was performed for TPA data using JMP software (JMP 10.2, SAS Inst.,).

## 3 Results and Discussion

### 3.1 Differential scanning calorimetry

**Figure 1:**
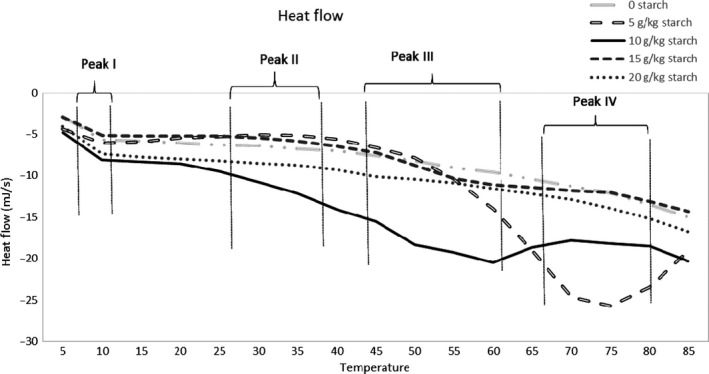
Differential scanning calorimetry (DSC) thermograms of protein pastes developed using increasing amounts of starch

Thermal changes induced in protein groups in myofibrillar protein gels containing increasing amounts of starch are presented in Figure 1 and Table 1. Myosin and actin are the main protein groups in myofibrillar protein and are responsible for cross‐linking and entrapping gel constituents. Starch as an additive may affect actin and myosin thermostability and denaturation enthalpies due to its ability to absorb and hold water as well as its gelation properties on its own. Peak I represents the denaturation and aggregation of myosin head, which was similar between gels containing different amounts of starch. Denaturation started around 6°C for myosin head, where the greatest amount (*p* < .05) of energy required to unfold the molecule was seen in gels containing 5 and 10 g starch/kg protein paste. Myosin tail is relatively less thermo‐susceptible than the head. Denaturation began around 26–33°C and enthalpy was increased (*p* < .05) with the addition of 5 g starch/kg protein paste. Peaks III and IV show that actin and sarcoplasmic protein unfolding temperatures, both at onset and maximum temperature required for denaturation, were similar between gel formulae; however, the energy required for denaturation was much higher (*p* < .05) for gels containing 5 g starch/kg protein paste.

**Table 1:** Differential scanning calorimetry (DSC) measurements (temperature onset (*T*_onset_), maximum temperature (*T*_max_), and net enthalpy) of isoelectrically recovered black bullhead catfish protein pastes developed using different amounts of starch (0, 5, 10, 15, 20 g/kg protein paste)

Starch (g/kg)	Peak I			Peak II		
	*T*_onset_ (^o^C)	*T*_max_ (^o^C)	Enthalpy (J/g)	*T*_onset_ (^o^C)	*T*_max_ (^o^C)	Enthalpy (J/g)
0	6.08 ± 0.31^ab^	11.10 ± 0.79^a^	1.79 ± 0.20^b^	26.26 ± 4.53	32.67 ± 4.19^b^	0.35 ± 0.09^c^
5	5.50 ± 0.26^b^	9.11 ± 0.71^b^	2.51 ± 0.11^a^	33.94 ± 5.72	41.55 ± 5.04^a^	1.95 ± 0.14^a^
10	6.28 ± 0.30^a^	10.76 ± 0.40^ab^	2.49 ± 0.32^a^	29.08 ± 1.51	35.93 ± 2.93^ab^	0.63 ± 0.17^bc^
15	5.98 ± 0.36^ab^	10.70 ± 0.54^ab^	1.67 ± 0.25^b^	26.96 ± 3.36	35.69 ± 2.40^ab^	1.02 ± 0.29^b^
20	6.01 ± 0.32^ab^	11.11 ± 1.68^a^	1.3 ± 0.12^b^	25.67 ± 2.80	33.31 ± 4.00^ab^	0.51 ± 0.16^c^
	Peak III			Peak IV		
0	54.38 ± 1.83^ab^	57.43 ± 1.26^bc^	0.22 ± 0.09^b^	71.47 ± 2.53	80.30 ± 1.69	0.27 ± 0.05^c^
5	56.4 ± 2.97^a^	69.36 ± 4.92^a^	62.40 ± 17.77^a^	70.18 ± 6.93	79.41 ± 5.41	8.26 ± 2.09^a^
10	55.02 ± 3.37^ab^	57.95 ± 2.85^bc^	1.74 ± 0.48^b^	74.83 ± 2.58	79.59 ± 3.95	0.55 ± 0.10^c^
15	51.51 ± 2.36^b^	64.56 ± 8.15^ab^	4.09 ± 1.09^b^	68.50 ± 4.06	73.53 ± 2.57	3.04 ± 0.34^b^
20	43.56 ± 0.21^c^	51.64 ± 4.15^c^	0.47 ± 0.11^b^	69.16 ± 2.01	74.67 ± 1.51	1.13 ± 0.25^bc^

Peak I and II represent the heat flow required to unfold myosin head and tail, respectively. Peak III and IV represent the heat flow required to unfold actin and sarcoplasmic protein, respectively. Data are given as mean ± standard deviation.

^a,b,c^Mean values with different letters are significantly different (Tukey's Honestly Significant Difference test, *p* < .05).

The binding effects of starch are highly dependent on the composition of the gel constituents as well as the type of starch incorporated into the protein paste. For example, amylose and amylopectin, the major components of starch, have different effects on protein gelation. Amylopectin is the main contributor to gel network formation in protein gels and increases gel strength and viscosity, whereas amylose does not show a synergistic effect in combination with protein (Joshi et al., 2013). Modified potato starch used in this study has 76% amylopectin, which is more than other starch sources. Granule size is also an important indicator of potential for swelling and is larger in potato starch compared to others like corn and lentil starch (Joshi et al., 2013). This may be the reason why a very small amount of starch, such as 5 g/kg protein paste, was able to affect protein unfolding and aggregation curves as shown by the high enthalpies (myosin tail, actin, and sarcoplasmic protein) when a very small amount of starch (5 g/kg protein paste) was added to protein paste. It is possible that the high swelling and water absorption properties of the potato starch granules increased the thermal stability of protein fractions without dominating or competing with protein groups for water absorption.

Starch granules are more thermostable and begin gelling after the protein groups (Kong, Ogawa, & Iso, 1999). Both protein and starch compete for the water in the gel, and since protein starts cross‐linking earlier, some portion of starch gelation is prevented by either lack of water or due to it being entrapped by the protein network (Hunt, Getty, & Park, 2009). Therefore, the lower thermal stability displayed by the enthalpies (myosin tail, actin, sarcoplasmic proteins) of gels containing higher amounts of starch (10, 15, 20 g/kg protein paste) may be due to the increased amylose and amylopectin in the paste not being able to gelatinize. Overall, the maximum amount of starch to be included in protein gels for avoiding increased thermal susceptibility depends greatly on the type and concentration of protein, starch, and the other additives in the paste, as well as the ionic strength, heating rate, and pH of the environment.

### 3.2 Expressible water content

**Figure 2:**
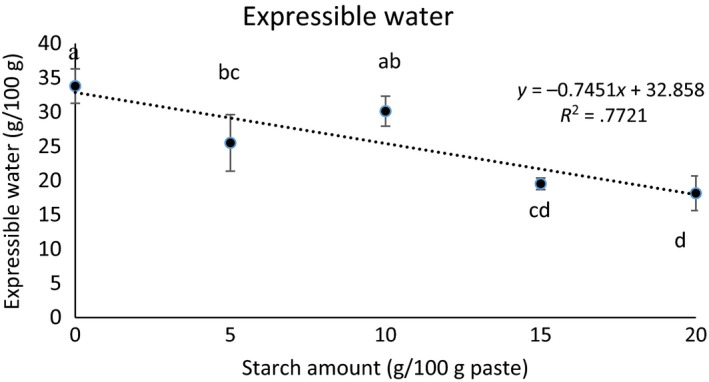
Expressible water (g/100 g) content of protein pastes containing increasing amounts of starch^a,b,c,d^. Mean values with different letters are significantly different

Expressible water content is an important measure that shows how well the gel had formed around water molecules, entrapping liquid constituents. Expressible water content decreased as starch amount increased (*p* < .05) due to the swelling of starch granules as expected (Figure 2). It is interesting to note that expressible water content was significantly lower (*p* < .05) in gels with no starch or 10 g starch/kg protein paste. The low expressible water content associated with these gels may be attributed to the calcium–starch interaction. Calcium binds to amino acids at the carboxyl side chains (Hauschka, Lian, & Gallop, 1975). Starch also forms hydrogen bonds at the carboxyl end of the proteins and may compete with calcium during gelation of calcium‐enhanced protein gels (Sanoja, Ruiz, Guyot, & Sanches, 2005). Moreover, increased calcium content is directly related to the increased swelling of protein gels and decreased elasticity and viscosity (Sanoja et al., 2005). Therefore, calcium competing with starch for both water and active binding sites of proteins was likely responsible for the functional and textural differences of gels having no starch or the lowest amount (5 g starch/kg protein paste) starch. It is possible that 10 g starch/kg protein paste starch interfered with the calcium–protein binding; however, the amount of starch was not enough to display its gel strengthening or water‐withholding properties. Therefore, higher amounts of starch (15, 20 g/kg protein paste) showed lower expressible water content.

### 3.3 Textural properties

**Figure 3:**
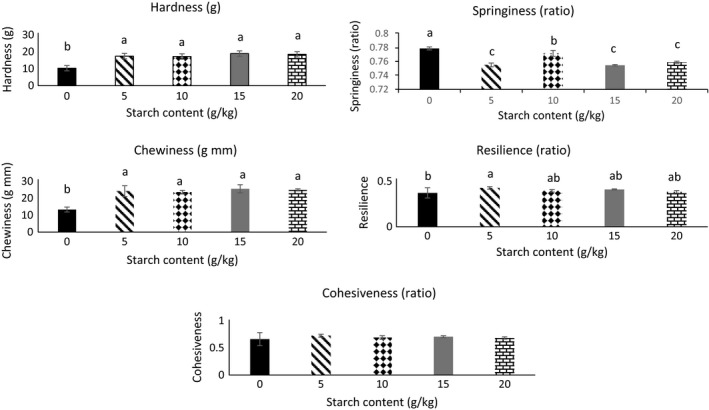
Texture profile analysis of gels containing increasing amounts of starch. Data are given as mean ± standard deviation. ^a, b, c^ Indicates differences between gels containing 0, 5, 10, 15, 20 g starch/kg protein paste)

Textural properties (hardness, springiness, cohesiveness, chewiness, and resilience) of the gels containing increasing amounts of starch are shown in Figure 3. For all concentrations, gels containing starch were harder, chewier, and less springy (*p* < .05) than gels without starch. The addition of 5 g starch/kg protein paste starch resulted in more resilient gels (*p* < .05); however, cohesiveness did not change with the addition of starch (*p* > .05), regardless of concentration. Increased calcium content has been linked to increased endogenous TGase activity and may be reason the “no starch gels” formed a consistent network. Calcium increased thermo‐stability of the protein gels by binding to the free carboxylic groups of aspartic and glutamic acids, thus triggering cross‐linking of proteins (Yang, Luan, Ashton, Gorczyca, & Kasapis, 2014).

**Figure 4:**
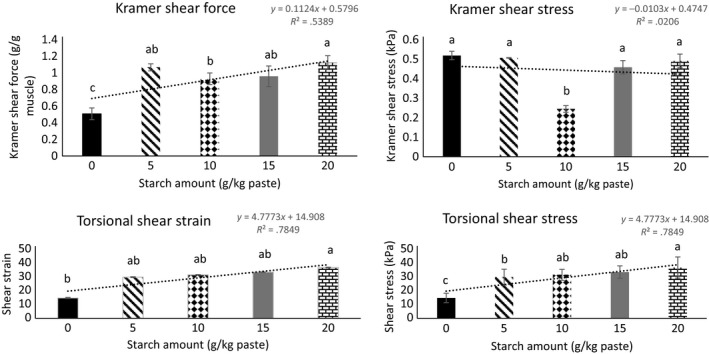
Texture analyses of gels containing increasing amounts of starch.^a, b, c^ Indicates differences between gels containing 0, 5, 10, 15, 20 g starch/kg protein paste)

Kramer shear force and stress, and torsional shear stress and strain of gels is another indicator of gel firmness and resistance to deformation both when the force is applied at the same plane of the product as well as at axial fracture. Linear regression indicated that torsional shear stress and strain increased as the concentration of starch increased (*R* ^*2*^ = .79 and .79, respectively) from 0 to 20 g starch/kg protein paste (Figure 4). These results are consistent with the expressible water data and shows that starch increases swelling of muscle fibers and water with holding capacity. Calcium‐enhanced protein is likely to produce more uniform gels due to increased cross‐linking interaction of myosin and actin. The effects of mineral and starch concentrations on final gel properties may depend on the amino acid composition of the myofibrillar protein, the amylose to amylopectin ratio of the tested potato starch, and the gel development and cooking strategy applied (Joshi et al., 2013).

### 3.4 Color

**Figure 5:**
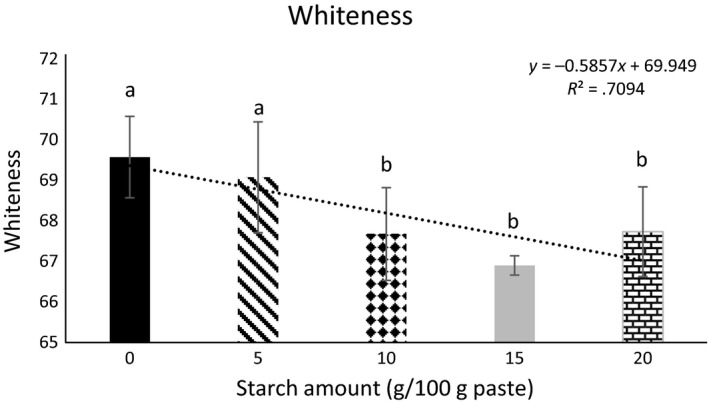
Whiteness of recovered protein gels containing increasing amounts of starch. ^a, b, c^ Indicates differences between gels containing 0, 5, 10, 15, 20 g starch/kg protein paste)

Color and whiteness properties of the protein gels containing increasing concentrations of starch are presented in Table 2 and Figure 5, respectively. Similar to the previous reports, the naturally lighter color of the calcium‐enhanced protein gels was adversely affected by the addition of greater amounts of starch (>10 g/kg protein paste) (Paker, Beamer, Jaczynski, & Matak, 2015; Paker and Matak, 2016). Although previous studies reported a trend toward green and blue hue, indicated by an increase in negative a* and b*, respectively, in this study a different color scheme was observed (Paker et al., 2015). Addition of the visibly yellow potato starch increased yellowness (*p* < .05) of gels as indicated by b* when compared to the gels containing no starch. This is consistent with previous results where starch‐containing gels were more yellow than gels with no starch (Paker et al., 2013a). The increased redness (a*) in the starch‐containing samples (*p* < .05) may be a result of amylose leakage from the granule which would contribute an opaque color (Chai & Park, 2007). When starch granules swell, amylose will leak into the protein paste where it will align and link itself with the protein. Upon cooking, amylose will precipitate which will not only strengthen the gel but also influence the final color (Chai & Park, 2007). Amylose leakage will lessen with greater concentrations of starch, which is likely why softer and darker gels are observed in gels containing higher concentrations of starch (Chai & Park, 2007; Giunee, Feeney, Auty, & Fox, 2002). It is possible that the amount of starch used in this study as an additive in myofibrillar protein gels was too small for differences to be observed. The amylose content may not have been adequate to improve gel color, thus there was a decrease in whiteness with the addition of potato starch.

**Table 2:** Color properties of recovered black bullhead catfish protein gels containing different starch amounts (0, 5, 10, 15, 20 g/kg protein paste), where L* indicates lightness (scale: 0–100), a* measures the intensity of red color (scale: −60 to +60), and b* shows the intensity of yellow color (scale: −60 to +60)

Starch (g/kg paste)	L*	a*	b*
0	70.88 ± 1.00^a^	−0.16 ± 0.07^b^	8.77 ± 0.49^b^
5	70.69 ± 1.56^a^	−0.33 ± 0.46^b^	9.79 ± 0.65^a^
10	69.09 ± 1.20^b^	−0.16 ± 0.14^b^	9.43 ± 0.29^a^
15	67.96 ± 1.12^b^	0.26 ± 0.08^a^	9.94 ± 0.22^a^
20	69.14 ± 1.00^b^	0.27 ± 0.32^a^	9.38 ± 0.88^a^

Data are given as mean ± standard deviation.

^a,b^Mean values with different letters are significantly different (Tukey's Honestly Significant Difference test, *p* < .05).

## 4 Conclusions

Overall, gels were harder, chewier, firmer, and more resistant to axial deformation (*p* < .05) when starch was added to protein gels. Textural properties (hardness, springiness, and chewiness) did not change significantly (*p* > .05) with increasing amounts of starch which may be due to the interactions and competition between protein, starch, and calcium to bind water in the paste. Gels got darker, redder, and more yellow with increasing amounts of starch. Results showed no benefit in increasing starch concentration in gels beyond 5 g starch/kg protein paste.

## Conflict of Interest

None declared.
